# Monoacylated Cellular Prion Proteins Reduce Amyloid-β-Induced Activation of Cytoplasmic Phospholipase A_2_ and Synapse Damage

**DOI:** 10.3390/biology4020367

**Published:** 2015-06-02

**Authors:** Ewan West, Craig Osborne, William Nolan, Clive Bate

**Affiliations:** Department of Pathology and Pathogen Biology, Royal Veterinary College, Hawkshead Lane, North Mymms, Herts AL97TA, UK; E-Mails: ejwest@rvc.ac.uk (E.W.); cosborne@rvc.ac.uk (C.O.); wnolan@rvc.ac.uk (W.N.)

**Keywords:** Alzheimer’s disease, amyloid-β, glycosylphosphatidylinositols, prion, synapses, synaptophysin

## Abstract

Alzheimer’s disease (AD) is a progressive neurodegenerative disease characterized by the accumulation of amyloid-β (Aβ) and the loss of synapses. Aggregation of the cellular prion protein (PrP^C^) by Aβ oligomers induced synapse damage in cultured neurons. PrP^C^ is attached to membranes via a glycosylphosphatidylinositol (GPI) anchor, the composition of which affects protein targeting and cell signaling. Monoacylated PrP^C^ incorporated into neurons bound “natural Aβ”, sequestering Aβ outside lipid rafts and preventing its accumulation at synapses. The presence of monoacylated PrP^C^ reduced the Aβ-induced activation of cytoplasmic phospholipase A_2_ (cPLA_2_) and Aβ-induced synapse damage. This protective effect was stimulus specific, as treated neurons remained sensitive to α-synuclein, a protein associated with synapse damage in Parkinson’s disease. In synaptosomes, the aggregation of PrP^C^ by Aβ oligomers triggered the formation of a signaling complex containing the cPLA_2_.a process, disrupted by monoacylated PrP^C^. We propose that monoacylated PrP^C^ acts as a molecular sponge, binding Aβ oligomers at the neuronal perikarya without activating cPLA_2_ or triggering synapse damage.

## 1. Introduction

Alzheimer’s disease (AD) is a complex neurological disorder that is characterized by a progressive dementia as a consequence of synapse failure [1]. The amyloid hypothesis of AD pathogenesis maintains that the primary event is the cleavage of the amyloid precursor protein by β- and γ-secretases into toxic amyloid-β (Aβ) fragments [2]. The accumulation of Aβ peptides, including *C*-terminal fragments of 42 residues (Aβ_42_), is thought to cause the abnormal phosphorylation of tau, synapse dysfunction and ultimately the clinical symptoms of AD. Aβ_42_ self-aggregates and is found in forms ranging from small soluble oligomers to much larger fibrils and plaques. The soluble Aβ oligomers that can diffuse throughout the brain are regarded as the most potent neurotoxins rather than Aβ fibrils or Aβ plaques [3,4]. For these studies, conditioned media from 7PA2 cells (7PA2-CM) containing natural Aβ oligomers [5] that have similar properties, including potency and stability, as the Aβ oligomers found within the cerebrospinal fluid of Alzheimer’s patients [6] were used.

The degree of dementia in AD correlates closely with the loss of synaptic proteins [7,8]. The process of AD-related synapse damage was examined by incubating cultured neurons with Aβ oligomers. Synaptic density in these neurons was determined by measuring the amounts of synaptophysin, a pre-synaptic membrane protein [9], using an enzyme-linked immunoassay (ELISA) [10]. The addition of Aβ reduced the synaptophysin content of cultured neurons indicative of synapse damage [10]. The loss of synaptophysin from neuronal cultures was accompanied by the loss of other synaptic proteins such as synapsin-1 and vesicle-associated membrane protein (VAMP)-1 [11]. This highly reproducible system was used to examine Aβ-induced synapse damage as a model of the synapse damage that occurs in AD.

Soluble Aβ oligomers are thought to bind to neurons in a receptor-mediated process. The identification of disease-relevant Aβ receptors remains controversial, as Aβ binds to many proteins, including the amyloid precursor protein [12], the receptor for advanced glycation end products (RAGE) [13], the p75 neurotrophin receptor [14], and metabotropic glutamate receptors [15]. Recently, the cellular prion protein (PrP^C^) was identified as a receptor that mediates Aβ-induced synapse dysfunction [16]. PrP^C^ is expressed at high levels within synapses [17] and aggregation of PrP^C^ by Aβ oligomers results in the activation of cytoplasmic phospholipase A_2_ (cPLA_2_) and synapse damage [11]. PrP^C^ is anchored to cell membranes by a glycosylphosphatidylinositol (GPI) anchor [18]. Since PrP^C^-mediated cell signaling was dependent upon the composition of the GPI anchor [19], the effects of PrP^C^ with a modified GPI anchor on Aβ-induced synapse damage was examined. We show that Aβ oligomers bind to PrP^C^ with a monoacylated GPI anchor (monoacylated PrP^C^). Pre-treatment of neurons with monoacylated PrP^C^ significantly reduced the Aβ-induced activation of cPLA_2_ and protected neurons against Aβ-induced synapse damage.

## 2. Experimental Section

**Primary neuronal cultures**: Cortical neurons were prepared from the brains of day 15.5 murine embryos derived from Prnp wild type^(+/+)^ or Prnp knockout^(0/0)^ mice. After mechanical dissociation, neurons were plated at 2 × 10^5^ cells/well in 48 well plates (pre-coated with poly-L-lysine) in Ham’s F12 containing 5% fetal calf serum for 2 h. Cultures were shaken (600 r.p.m for 5 min) and non-adherent cells removed by 3 washes in PBS. Neurons were grown in neurobasal medium containing B27 components and nerve growth factor (5 ng/mL) for 10 days. Immunohistochemistry showed that 95% of the cells were neurofilament positive. To determine cell viability thiazolyl blue tetrazolium bromide (MTT) was added to neuronal cultures at a final concentration of 50 µM for 3 h at 37 °C. The supernatant was removed, the formazan product solubilized in 200 μL of dimethyl sulfoxide, transferred to an immunoassay plate and absorbance read at 595 nm. Neuronal survival was calculated with reference to untreated neurons (100% survival).

**Cell extracts**: Treated neurones were washed 3 times with PBS and homogenized in a buffer containing 150 mM NaCl, 10 mM Tris-HCl, pH 7.4, 10 mM EDTA, 0.2% SDS, mixed protease inhibitors (4-(2-aminoethyl)benzenesulfonyl flouride, Aprotinin, Leupeptin, Bestain, Pepstatin A and E-46) and a phosphatase inhibitor cocktail (PP1, PP2A, microcystin LR, cantharidin and p-bromotetramisole) (Sigma, Poole, UK) at 10^6^ cells/mL. Nuclei and cell debris was removed by centrifugation (300× *g* for 5 min).

**Isolation of synaptosomes**: Synaptosomes were prepared on a discontinuous Percoll gradient. Cortical neurons were homogenized at 4 °C in 1 mL of SED solution (0.32 M sucrose, 50 mM Tris-HCl, pH 7.2, 1 mM EDTA, and 1 mM dithiothreitol and centrifuged at 1000× *g* for 10 min). The supernatant was transferred to a 4-step gradient of 3, 7, 15, and 23% Percoll in SED solution and centrifuged at 16,000× *g* for 30 min at 4 °C. The synaptosome fractions were collected from the interface of the 15% and 23% Percoll steps, washed twice (16,000× *g* for 30 min at 4 °C) and suspended in extraction buffer (150 mM NaCl, 10 mM Tris-HCl pH 7.4, 10 mM EDTA, 0.2% SDS and mixed protease/phosphatase inhibitors).

**Isolation of DRMs**: These membranes were isolated by their insolubility in non-ionic detergents, as previously described [20]. Briefly, samples were homogenized in an ice-cold buffer containing 1% Triton X-100, 10 mM Tris-HCl, pH 7.2, 150 mM NaCl, 10 mM EDTA and mixed protease inhibitors and nuclei and large fragments were removed by centrifugation (300× *g* for 5 min at 4 °C). The supernatant was incubated on ice (4 °C) for 1 h and centrifuged (16,000× *g* for 30 min at 4 °C). The supernatant was reserved as the detergent soluble membrane (DSM), while the insoluble pellet was homogenized in an extraction buffer containing 10 mM Tris-HCL, pH 7.4, 150 mM NaCl, 10 mM EDTA, 0.5% Nonidet P-40, 0.5% sodium deoxycholate, 0.2% SDS and mixed protease inhibitors at 10^6^ cells/mL, centrifuged (10 min at 16,000× *g*) and the soluble material was reserved as the DRM fraction.

**Western Blotting**: Samples were mixed with Laemmli buffer containing β-mercaptoethanol, heated to 95°C for 5 min and proteins were separated by electrophoresis on 15% polyacrylamide gels (PAGE). Proteins were transferred onto a Hybond-P PVDF membrane by semi-dry blotting. Membranes were blocked using 10% milk powder; synapsin-1 was detected with goat polyclonal (Santa Crux Biotech, London, UK), vesicle-associated membrane protein (VAMP)-1 with mAb 4H302 (Abcam, Cambridge, UK), rabbit polyclonal antibodies to caveolin (Upstate, Damstadt, Germany), cPLA_2_ with mAb CH-7 (Upstate) and PrP^C^ by mAb 4F2 (Jaques Grassi, Parus, France); these were visualized using a combination of biotinylated anti-mouse/goat/rat/rabbit IgG (Sigma), extravidin-peroxidase and enhanced chemiluminescence.

**Isolation of GPI anchored proteins**: PrP^C^ and Thy-1 were isolated from GT1 murine neuronal cells, as previously described [21]. Briefly, membranes were homogenized in a buffer containing 10 mM Tris-HCl pH 7.4, 100 mM NaCl, 10 mM EDTA, 0.5% Nonidet P-40, 0.5% sodium deoxycholate and mixed protease inhibitors (as above) and passed over affinity columns loaded with mAbs to PrP^C^ (ICSM18) or anti-Thy-1 (Serotec, Kidlington, UK). PrP^C^ and Thy-1 was eluted using glycine-HCl at pH 2.7, neutralized with 1 M Tris pH 7.4 and desalted (3 kDa filter, Sartorius). Proteins were digested with 100 units/mL bee venom phospholipase A_2_ (PLA_2_) (Sigma) to generate monoacylated PrP^C^ and monoacylated Thy-1 (37 °C for 1 h) and isolated via reverse phase chromatography on C18 columns (Waters) using a gradient of propanol in water. PrP containing fractions were pooled, desalted, and concentrated. For high performance thin-layer chromatography (HPTLC) analysis, samples were dissolved in ethanol and separated on silica gel 60 plates using a mixture of chloroform/methanol/water (10/10/3 v/v/v). Plates were soaked in 0.1% polyisobutyl methacrylate in hexane, dried, and blocked with 5% milk powder. PrP^C^ was detected with mAb 4F2. For bioassays, samples were solubilized in culture medium by sonication.

**Synaptophysin ELISA**: The amounts of synaptophysin in neurons were measured by ELISA [22]. Maxisorb immunoplates (Nunc, Roskilde, Denmark) were coated with a mouse monoclonal antibody (mAb) to synaptophysin MAB368 (Millipore, Damstadt, Germany). Samples were applied and bound, synaptophysin was detected using rabbit polyclonal anti-synaptophysin (Abcam) followed by a biotinylated anti-rabbit IgG, extravidin-alkaline phosphatase and 1 mg/mL 4-nitrophenol phosphate (Sigma). Absorbance was measured on a microplate reader at 405 nm and the synaptophysin content calculated. Samples were expressed as “units synaptophysin”, where 100 units was the amount of synaptophysin in 10^6^ control neurons.

**cPLA_2_ ELISA**: The amounts of cPLA_2_ in extracts was measured by ELISA [21]. Maxisorb immunoplates were coated with 0.5 µg/mL of mouse mAb anti-cPLA_2_ (clone CH-7—Upstate) and blocked with 5% milk powder. Samples were incubated for 1 h and the amount of bound cPLA_2_ was detected using a goat polyclonal anti-cPLA_2_ (Santa-Cruz Biotech, London, UK) followed by biotinylated anti-goat IgG, extravidin-alkaline phosphatase and 1 mg/mL 4-nitrophenol phosphate. Absorbance was measured at 405 nm and the amount of cPLA_2_ protein expressed in units, 100 units = amount of cPLA_2_ in control preparations.

**Activated cPLA_2_ ELISA**: The activation of cPLA_2_ is accompanied by the phosphorylation of the 505 serine residue and can be measured by phospho-specific antibodies. Maxisorb immunoplates were coated with 100 nM mAb anti-cPLA_2_, clone CH-7 (Upstate) and blocked with 10% milk powder. Samples were incubated for 1 h and the amount of activated cPLA_2_ was detected using a rabbit polyclonal anti-phospho-cPLA_2_ (Cell Signaling Technology, Cambridge, UK), biotinylated anti-rabbit IgG, extravidin-alkaline phosphatase and 1 mg/mL 4-nitrophenyl phosphate. Absorbance was measured at 405 nm and the amounts of activated cPLA_2_ present were expressed as “units activated cPLA_2_”, where 100 units were defined as the amount of activated cPLA_2_ in control synaptosomes.

**PrP^C^ ELISA**: The amount of PrP^C^ in samples was determined by [10]. Maxisorb immunoplates were coated with mAb ICSM18 (Dr. Mourad Tayebi). Samples were added and bound PrP was detected with biotinylated mAb ICSM35 (Dr. Mourad Tayebi). Biotinylated mAb was detected using extravidin-alkaline phosphatase and 1 mg/mL 4-nitrophenyl phosphate. Absorbance was measured on a microplate reader at 405 nm and the amount of PrP in samples was calculated by reference to a standard curve of recombinant murine PrP (Prionics, London, UK).

**Preparation of Aβ-containing medium**: CHO cells stably transfected with a cDNA encoding APP_751_ (referred to as 7PA2 cells) were cultured in DMEM with 10% fetal calf serum as described [5]. Conditioned medium (CM) from these cells contains Aβ oligomers (7PA2-CM). CM from non-transfected CHO cells (CHO-CM) was used as controls. 7PA2-CM and CHO-CM were centrifuged at 100,000× *g* for 4 h at 4 °C to remove cell debris and then passed through a 50 kDa filter (Sartorius, Damstadt, Germany). 7PA2-CM contains Aβ monomers and low-n Aβ oligomers [5]. For immunoblot analysis, extracts were concentrated, mixed with an equal volume of 0.5% NP-40, 5 mM CHAPS, 50 mM Tris, pH 7.4 and separated by electrophoresis using Novex, Triz-glycine native running buffer (Life technologies, Paisley, UK). Proteins were transferred onto a PVDF membrane by semi-dry blotting and blocked using 10% milk powder. Aβ was detected by incubation with mAb 6E10 (Covance, Maidenhead, UK), biotinylated anti-mouse IgG, extravidin-peroxidase and enhanced chemiluminescence. The amounts of Aβ_42_ in preparations were determined by ELISA.

**Immunodepletions**: 7PA2-CM were incubated with 0.1 μg/mL mAb 4G8 (reactive with amino acids 17–24 of Aβ) or isotype controls (mock-depletion) and incubated at 4 °C on rollers for 24 h. Protein G microbeads were added (10 µL/mL) (Sigma) for 2 h and protein G bound-antibody complexes removed by centrifugation and filtration.

**Sample preparation for end-specific ELISAs**: To detach Aβ_42_ from cellular components that could occlude specific epitopes samples (50 µL) were mixed with 250 µL of 70% formic acid and sonicated. A 50 µL aliquot was added to 50 µL of 10M Tris-HCl with protease inhibitors (as above) and sonicated before addition to ELISA.

**Aβ_42_ ELISA**: Maxisorb immunoplates were coated with mAb 4G8 (epitope 17–24) (Covance). Plates were blocked with 5% milk powder and samples were applied. The detection antibody was an Aβ_42_ selective rabbit mAb BA3-9 (Covance) followed by biotinylated anti-rabbit IgG and extravidin alkaline phosphatase. Total Aβ was visualized by addition of 1mg/mL 4-nitrophenol phosphate solution and optical density was read in a spectrophotometer at 405 nm.

**PrP^C^-Aβ ELISA**: Maxisorb immunoplates were coated with 10 nM PrP^C^, monoacylated PrP^C^ or monoacylated Thy-1 and blocked with 5% milk powder. Samples were added for 1 h and bound Aβ was detected with biotinylated mAb 4G8 (epitope 17–24 of Aβ) (Covance), followed by extravidin-alkaline phosphatase and 1 mg/mL 4-nitrophenol phosphate solution. Optical density was read in a spectrophotometer at 405 nm.

**Peptides**: Recombinant human αSN was obtained from Sigma. Peptides were thawed on the day of use and mixed in neurobasal medium containing B27. Mixtures were subjected to sonication and vigorous shaking (disruptor genie, full power for 10 min) before they were added to neurons.

**Statistical Analysis**: Comparison of treatment effects was carried out using Student’s paired *t*-tests.

## 3. Results and Discussion

**Figure 1:**
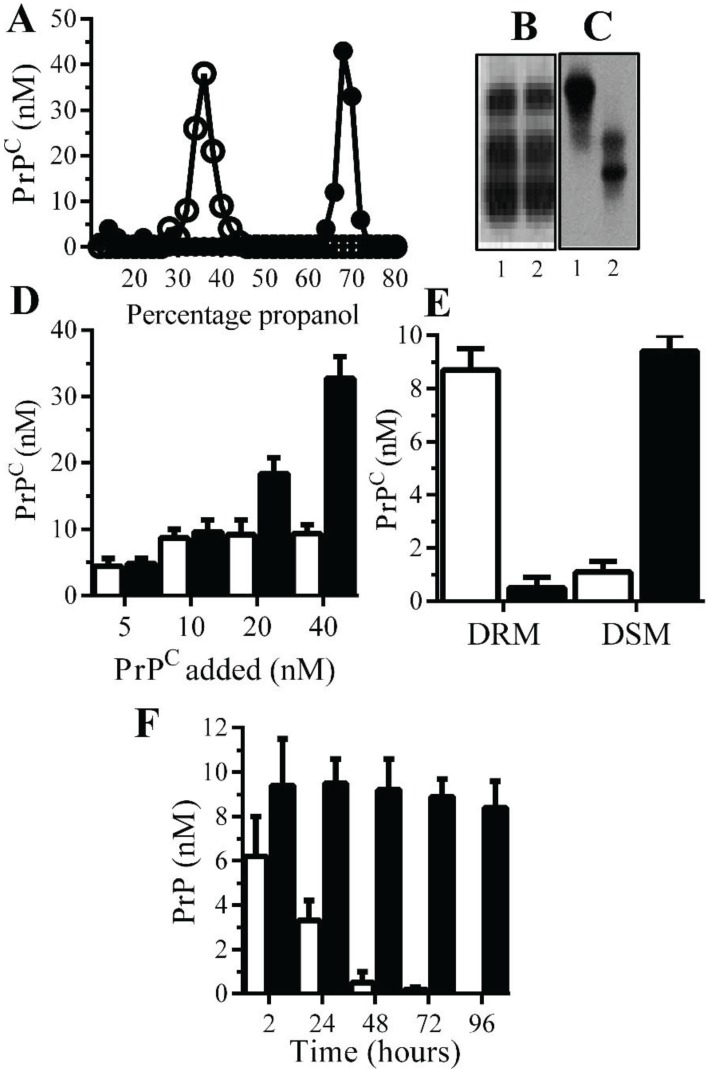
Monoacylated PrP^C^ is expressed in neurons: (**A**) The concentrations of PrP^C^ (●) or monoacylated PrP^C^ (○) in fractions eluted from C18 columns. Values are means of duplicates; PrP^C^ (1) and monoacylated PrP^C^ (2) separated by PAGE (**B**) or HPTLC (**C**). (**D**) The concentrations of PrP^C^ in Prnp^(0/0)^ neurons treated with PrP^C^ (□) and monoacylated PrP^C^ (■), as shown for 2 h. Values are means ± SD from triplicate experiments performed four times (*n* = 12). (**E**) The concentrations of PrP^C^ (□) and monoacylated PrP^C^ (■) in DRM (rafts) or DSMs in Prnp^(0/0)^ neurons pulsed with 10 nM PrP^C^ preparations for 2 h. Values are means ± SD from triplicate experiments performed four times (*n* = 12). (**F**) The concentrations of PrP^C^ (□) and monoacylated PrP^C^ (■) in Prnp^(0/0)^ neurons at different time periods after being pulsed with 10 nM PrP^C^ preparations. Values are means ± SD from triplicate experiments performed four times (*n* = 12).

**Monoacylated PrP^C^ is stable within neuronal membranes**: Monoacylated PrP^C^ eluted from C18 columns at lower concentrations of propanol than PrP^C^ (Figure 1A). Western blots demonstrated that there was no obvious difference in the molecular weight of PrP^C^ and monoacylated PrP^C^ (Figure 1B), which is consistent with the loss of an acyl chain with a molecular mass of ~0.2 kDa. The loss of a hydrophobic acyl chain resulted in monoacylated PrP^C^ migrating differently from PrP^C^ in HPTLC (Figure 1C). Many GPI-anchored proteins bind to recipient cells [23] and both PrP^C^ and monoacylated PrP^C^ bound to Prnp^(0/0)^ neurons in neurons in a dose-dependent manner (Figure 1D). Whereas PrP^C^ was found within DRMs (lipid rafts), the monoacylated PrP^C^ was found within DSMs (normal cell membrane) [21] (Figure 1E). In neurons from Prnp^(0/0)^, mice PrP^C^ had a half-life of less than 24 h in accordance with previous reports [24], whereas monoacylated PrP^C^ remained in neurons far longer and had a half-life of greater than four days (Figure 1F).

**Figure 2:**
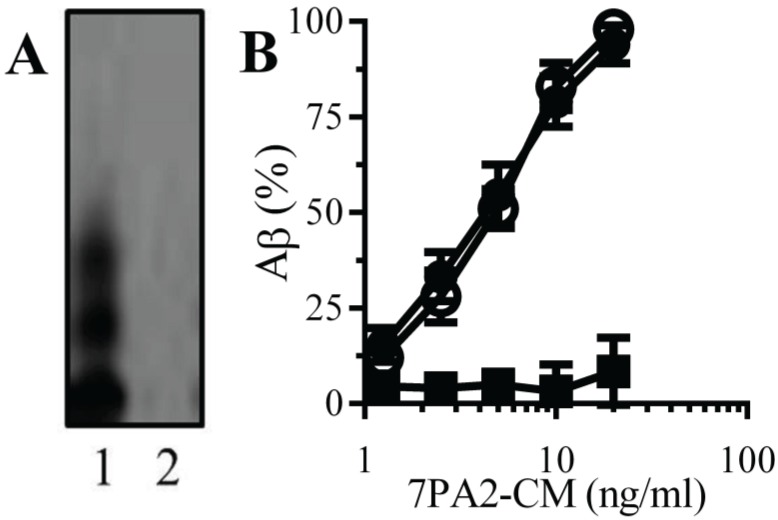
Aβ binds to monoacylated PrP^C^: (**A**) Immunoblots showing forms of Aβ in 7PA2-CM (1) and CHO-CM (2). (**B**) The amounts of Aβ detected in immunoplates coated with 10 nM PrP^C^ (●), 10 nM monoacylated PrP^C^ (○) or 10 nM monoacylated Thy-1 (■) and incubated with 7PA2-CM as shown. Values are means ± SD from triplicate experiments performed 4 times (*n* = 12).

**Natural Aβ binds to monoacylated PrP^C^**: PrP^C^ acts as a receptor for Aβ and mediated Aβ-induced synapse damage [11,16]. Since the presence of a GPI anchor affected the structure of some proteins [25], the binding of Aβ to monoacylated PrP^C^ was examined. An immunoblot showed that 7PA2-CM contained several forms of Aβ that were not found in CHO-CM (Figure 2A). When immunoplates coated with 10 nM PrP^C^, 10 nM monoacylated PrP^C^ or monoacylated Thy-1 PrP^C^ were incubated with 7PA2-CM, Aβ bound to both PrP^C^ and monoacylated PrP^C^ without binding to monoacylated Thy-1 (Figure 2B).

**Figure 3:**
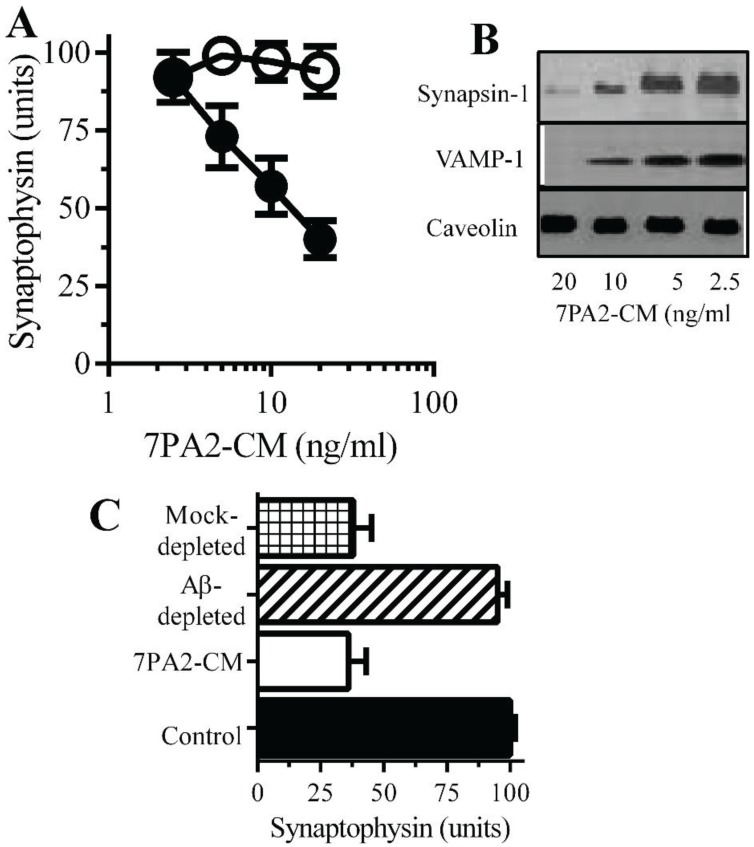
Aβ oligomers cause synapse damage in neurons: (**A**) The amounts of synaptophysin in cultured neurons incubated with 7PA2-CM (●) or CHO-CM (○) for 24 h. Values are means ± SD from triplicate experiments performed 4 times (*n* = 12). (**B**) Immunoblots showing the amount of synapsin-1, VAMP-1 and caveolin in neurons incubated with 7PA2-CM for 24 h. (**C**) The amounts of synaptophysin in neurons incubated with control medium (■), 7PA2-CM (□), Aβ-depleted 7PA2-CM (striped bar) or mock-depleted 7PA2-CM (hatched bar) for 24 h. Values are means ± SD from triplicate experiments performed four times (*n* = 12).

**Aβ oligomers trigger synapse damage**: Since the loss of synaptic proteins is a feature of AD that strongly correlates with cognitive decline [7,8] the amounts of synaptophysin in neurons incubated with Aβ was studied. 7PA2-CM, but not CHO-CM, reduced the synaptophysin content of neurons indicative of synapse damage (Figure 3A) [11]. Immunoblots showed that 7PA2-CM also caused the loss of synapsin-1 and VAMP-1 from cultured neurons but without affecting the amounts of caveolin (Figure 3B). The addition of CHO-CM to neurons did not significantly affect levels of synaptic proteins. These concentrations of 7PA2-CM did not significantly reduce cell viability as measured by the MTT method (98% ± 4% cell survival, compared to 100% ± 5%, *p* = 0.45, *n* = 9). The addition of 7PA2-CM that had been depleted of Aβ did not trigger the loss of synaptophysin from neurons (Figure 3C) indicating that Aβ was responsible for synapse damage.

**Figure 4:**
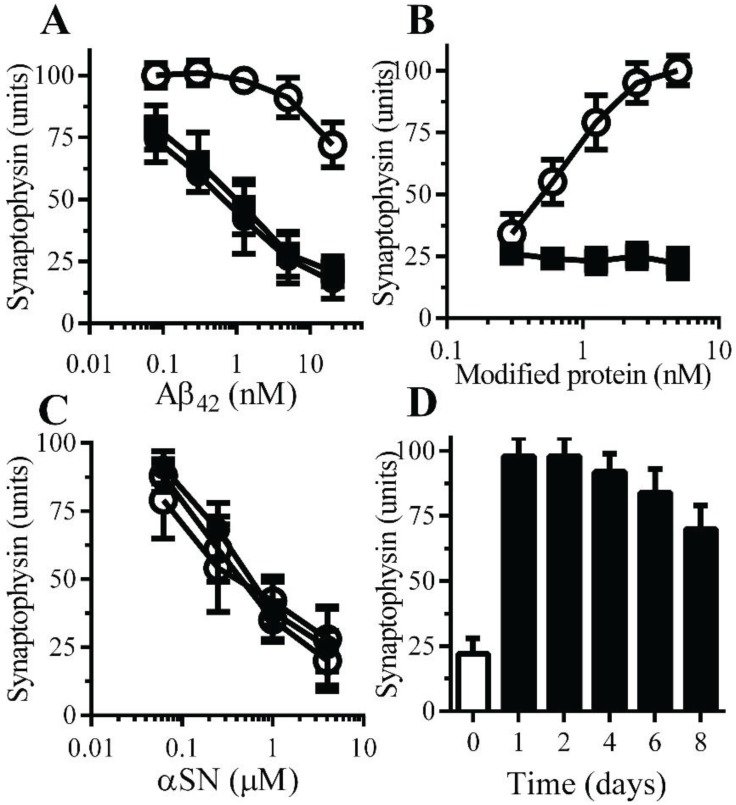
Monoacylated PrP^C^ protected neurons against Aβ-induced synapse damage: (**A**) The amounts of synaptophysin in neurons pre-treated with control medium (●), 10 nM monoacylated PrP^C^ (○) or 10 nM monoacylated Thy-1 (■) and incubated with Aβ_42_. Values are means ± SD, from triplicate experiments performed four times, *n* = 12. (**B**) The amounts of synaptophysin in neurons pre-treated with monoacylated PrP^C^ (○) or monoacylated Thy-1 (■) as shown and incubated with 10 nM Aβ_42_. Values are means ± SD, from triplicate experiments performed four times, *n* = 12; (**C**) The amounts of synaptophysin in neurons pre-treated with control medium (●), 10 nM monoacylated PrP^C^ (○) or 10 nM monoacylated Thy-1 (■) and incubated with αSN. Values are means ± SD, from triplicate experiments performed four times, *n* = 12. (**D**) The amounts of synaptophysin in neurons pre-treated with control medium (□) or 10 nM monoacylated PrP^C^ for different time periods as shown (■) and incubated with 10 nM Aβ_42_. Values are means ± SD, from triplicate experiments performed three times, *n* = 9.

**Monoacylated PrP^C^ reduced Aβ-induced synapse damage**: The polymorphic nature of Aβ aggregates indicates that there are disease-relevant conformational forms of Aβ, while other conformations are less toxic [26]. The possibility that it was mainly the non-toxic conformations of Aβ that bound to monoacylated PrP^C^ was tested by examining the effects of monoacylated PrP^C^ upon Aβ-induced synapse damage. The addition of either monoacylated PrP^C^ or monoacylated Thy-1 did not cause synapse damage as determined by the loss of synaptophysin from neurons. However, pre-treatment of neurons with 10 nM monoacylated PrP^C^, but not with 10 nM monoacylated Thy-1, reduced the Aβ-induced synapse damage (Figure 4A). The presence of monoacylated PrP^C^ protected neurons against Aβ-induced synapse damage in a dose-dependent manner (Figure 4B). The synapse damage in Parkinson’s disease (PD) and dementia with Lewy bodies is associated with the accumulation of α-synuclein (αSN) at synapses [27] and the addition of recombinant human αSN triggered synapse damage in neurons [28]. Pre-treatment of neurons with 10 nM monoacylated PrP^C^ did not affect αSN-induced synapse damage (Figure 4C). To determine whether the protective effect of monoacylated PrP^C^ was long lived, neurons were pulsed with 10 nM monoacylated PrP^C^ for 1 h and 10 nM Aβ_42_ was added at time points thereafter. Neurons treated with monoacylated PrP^C^ remained resistant to Aβ-induced synapse damage for eight days (Figure 4D).

**Figure 5:**
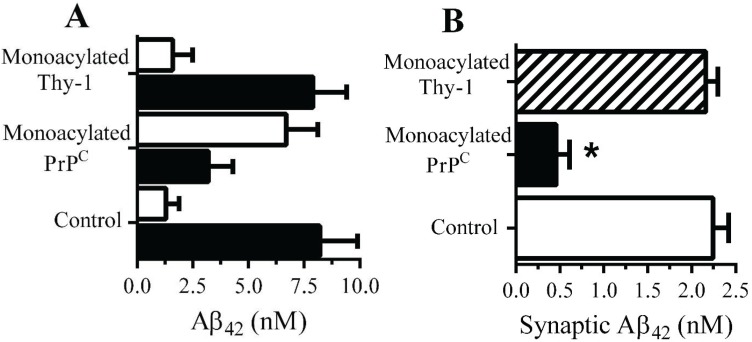
Monoacylated PrP^C^ reduced the accumulation of Aβ_42_ at synapses: (**A**) The concentrations of Aβ_42_ in DRMs (■) or DSMs (□) of neurons pre-treated with control medium, 10 nM monoacylated PrP^C^ or 10 nM monoacylated Thy-1 as shown and incubated with 10 nM Aβ_42_. Values are means ± SD from triplicate experiments performed three times (*n* = 9). (**B**) The concentrations of Aβ_42_ in synaptosomes derived from neurons pre-treated with control medium (□), 10 nM monoacylated PrP^C^ (■) or 10 nM monoacylated Thy-1 (striped bar) and incubated with 10 nM Aβ_42_ for 2 h. Values are means ± SD from triplicate experiment performed four times, *n* = 4.

**Monoacylated** **PrP^C^ reduced** **the accumulation of** **Aβ_42_ within synapses**: The presence of 10 nM monoacylated PrP^C^ on neurons did not affect the binding of Aβ to neurons; 2 h after the addition of 10 nM Aβ_42_ there were no significant differences between control and treated neurons (9.2 nM Aβ_42_ ± 0.8 compared with 8.9 nM ± 1, *n* = 9, *p* = 0.4). Whereas the majority of Aβ_42_ added to control neurons was found within DRMs (rafts), consistent with reports [29], in neurons pre-treated with 10 nM monoacylated-PrP^C^ significantly less Aβ_42_ was found within DRMs and more with the DSMs (Figure 5A). The targeting of Aβ_42_ to rafts may affect the subsequent trafficking of Aβ_42_, which accumulates within synapses in control neurons [11]. To determine whether Aβ_42_ had the same fate in treated neurons they were pulsed with 10 nM monoacylated PrP^C^ or monoacylated Thy-1 and incubated with 10 nM of Aβ_42_ for 2 h and synaptosomes isolated. Pre-treatment with monoacylated PrP^C^ significantly reduced the concentrations of Aβ_42_ found in synaptosomes compared to control neurons or neurons pre-treated with 10 nM monoacylated Thy-1 (Figure 5B).

**Figure 6:**
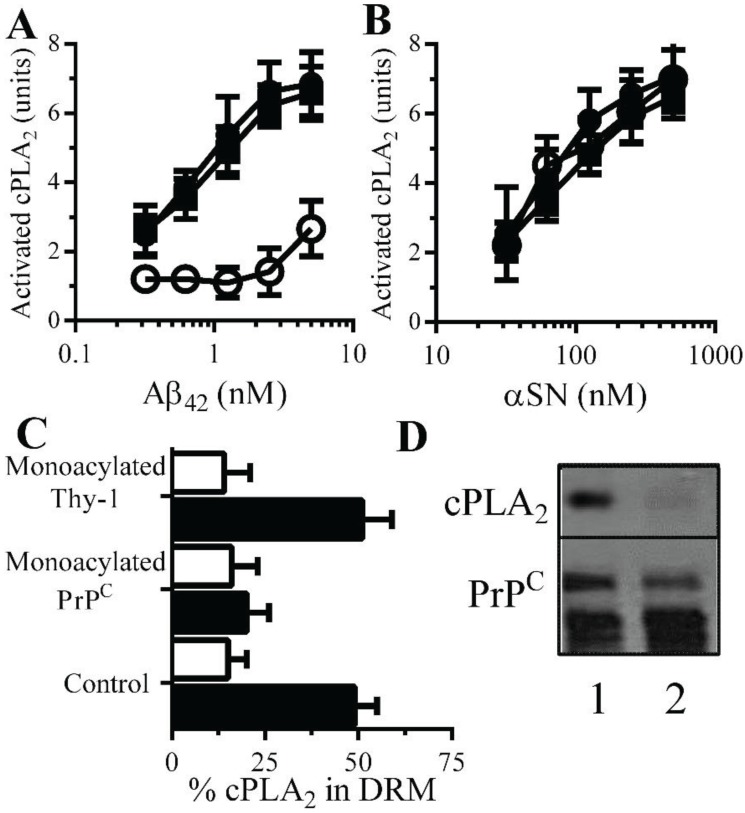
Monoacylated PrP^C^ reduced Aβ-induced activation of cPLA_2_ in synaptosomes: The amounts of activated cPLA_2_ in synaptosomes pre-treated with control medium (●), 10 nM monoacylated PrP^C^ (○) or 10 nM monoacylated Thy-1 (■) and incubated with Aβ_42_ (**A**) or αSN (**B**) for 1 h. Values are means ± SD from triplicate experiments performed three times (*n* = 9). (**C**) The amounts of cPLA_2_ in DRMs (rafts) from synaptosomes pre-treated with control medium, 10 nM monoacylated PrP^C^ or 10 nM monoacylated Thy-1 and incubated with control medium (□) or 10 nM Aβ_42_ (■) for 1 h. (**D**) Blot showing the amounts of cPLA_2_ and PrP^C^ in immunoprecipitates from synaptosomes treated with control medium (1) or 10 nM monoacylated PrP^C^ (2) and incubated with the PrP^C^-reactive mAb (4F2) for 1 h.

**Monoacylated** **PrP^C^ reduced** **Aβ-induced activation of cPLA_2_ in synapses**: There is evidence that Aβ-induced aberrant activation of cell signaling pathways is involved in synapse damage. Since Aβ activates cPLA_2_[10,30], and pharmacological inhibition of PLA_2_ protected against Aβ-induced synapse damage [10], the effect of monoacylated PrP^C^ on the activation of cPLA_2_ was examined. The amount of activated cPLA_2_ in synaptosomes was not affected by addition of 10 nM monoacylated PrP^C^ (108 units activated cPLA_2_ ± 12 compared with 100 units ± 14, *n* = 9, *p* = 0.4) or monoacylated Thy-1 (104 units activated cPLA_2_ ± 12 compared with 100 units ± 14, *n* = 9, *p* = 0.6). However, pre-treatment of neurons with 10 nM monoacylated PrP^C^, but not 10 nM monoacylated Thy-1, significantly reduced the Aβ-induced activation of cPLA_2_ in synaptosomes (Figure 6A). In contrast, pre-treatment with 10 nM monoacylated PrP^C^ did not affect αSN-induced activation of cPLA_2_ (Figure 6B). Activation of cPLA_2_ is associated with its translocation to specific membrane micro-domains by an N-terminal lipid-binding motif [31]. Sucrose density gradients showed that in synaptosomes, the addition of Aβ results in the migration of cPLA_2_ to DRMS [11]. In control synaptosomes, incubated with 1 nM Aβ_42_ approximately 50% of cPLA_2_ was found within DRMs (Figure 6C). In synaptosomes pre-treated with 10 nM monoacylated PrP^C^ and incubated with 1 nM Aβ_42_, significantly less cPLA_2_ was found within DRMs. The addition of 10 nM monoacylated Thy-1 did not affect the Aβ-induced translocation of cPLA_2_ to DRMs. The aggregation of PrP^C^ by Aβ oligomers results in the formation of a signaling complex containing cPLA_2_ [11]. To determine whether monoacylated PrP^C^ interfered with the formation of these complexes synaptosomes were pre-treated with 10 nM monoacylated PrP^C^ or 10 nM monoacylated Thy-1 and incubated with 10 nM mAb 4F2 for 1 h. The presence of monoacylated PrP^C^ resulted in complexes that did not contain cPLA_2_ (Figure 6D).

**Figure 7:**
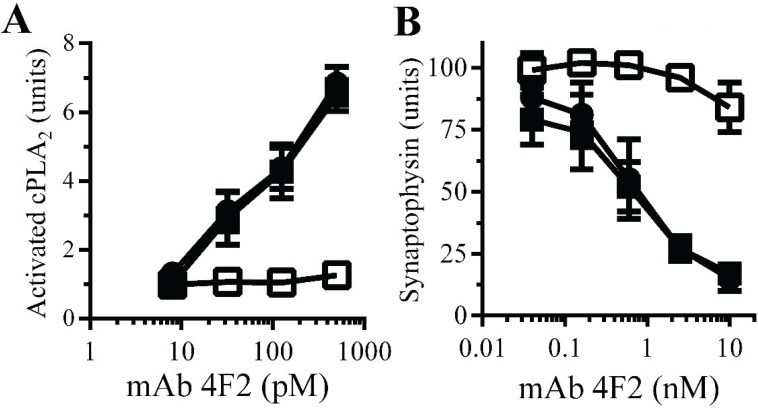
Monoacylated PrP^C^ reduced mAb 4F2-induced synapse damage: (**A**) The amounts of activated cPLA_2_ in synaptosomes pre-treated with control medium (●), 10 nM monoacylated PrP^C^ (□) or 10 nM monoacylated Thy-1 (■) and incubated with mAb 4F2. Values are means ± SD from triplicate experiments performed three times (*n* = 9). (**B**) The amounts of synaptophysin in neurons pre-treated with control medium (●), 10 nM monoacylated PrP^C^ (□) or 10 nM monoacylated Thy-1 (■), incubated with mAb 4F2. Values are means ± SD from triplicate experiments performed three times, *n* = 9.

**Monoacylated PrP^C^ reduced synapse damage induced by the PrP^C^-reactive mAb 4F2**: The observations that Aβ oligomers trigger neurodegeneration and that Aβ oligomers cross-link PrP^C^ at synapses [11] suggested that aggregation of PrP^C^ by Aβ oligomers. PrP^C^-reactive mAbs cause neurodegeneration *in vivo* [32] and trigger synapse damage *in vitro* [11]. The PrP^C^-reactive mAb 4F2 mimicked some of the effects of Aβ upon synaptosomes, including increasing the activation of cPLA_2_. Here we show that pre-treatment of synaptosomes with 10 nM monoacylated PrP^C^, but not monoacylated Thy-1, significantly reduced the mAb 4F2-induced activation of cPLA_2_ (Figure 7A). In addition, pre-treatment of neurons with 10 nM monoacylated PrP^C^, but not 10 nM monoacylated Thy-1, significantly reduced mAb 4F2-induced synapse damage (Figure 7B).

## 4. Discussion

PrP^C^ acts as a receptor for the Aβ oligomers that cause cognitive impairment in a model of AD [16] and mediated Aβ-induced synapse damage in cultured neurons [11]. The key finding of this study was that the presence of monoacylated PrP^C^ significantly reduced Aβ-induced synapse damage. The protective effects of monoacylated PrP^C^ were related to two interrelated activities, the disruption of Aβ-induced cell signaling and the sequestration of Aβ outside lipid rafts.

Although PrP^C^ is associated with several signaling pathways, it lacks a transmembrane component. The GPI attached to PrP^C^ targets the protein to lipid rafts [33] in which signaling complexes, often called signalosomes, assemble [34]. PrP^C^ is thought to act as a “scaffold protein” which organizes the composition and function of signalosomes. Observations that Aβ is found within lipid rafts [29] and that Aβ-induced synapse damage is sensitive to raft disruption [35] suggest that the events leading to synapse degeneration are initiated from within lipid rafts. Consistent with this theory, aggregation of PrP^C^ by Aβ oligomers induced activation of cPLA_2_ and led to synapse degeneration [11].

We demonstrate that the properties of monoacylated PrP^C^ were different from those of PrP^C^. Perhaps the key observation was that monoacylated PrP^C^ was not targeted to membrane rafts [21]. PrP^C^ is a recycling protein that like many raft-associated proteins traffics to and from the plasma membrane [36], whereas monoacylated PrP^C^ was found within the normal cell membrane. While the loss of an acyl chain from PrP^C^ affected membrane targeting it did not affect the binding of soluble Aβ suggesting that the protein structure was not altered. Therefore it was not surprising to find that in neurons decorated with monoacylated PrP^C^ a significant percentage of Aβ_42_ was found outside rafts consistent with the hypothesis that monoacylated PrP^C^ sequestered Aβ_42_ into non-signaling membrane domains. The targeting of proteins to rafts also affects the trafficking of proteins [37] and monoacylated PrP^C^ reduced the accumulation of Aβ at synapses. These results are consistent with the hypothesis that monoacylated PrP^C^ acts as a molecular sponge which adsorbs Aβ in specific cell compartments, in the process preventing Aβ binding to native PrP^C^ and triggering the raft-dependent signaling that leads to synapse damage.

The key finding, that neurons decorated with monoacylated PrP^C^ were less susceptible to Aβ-induced synapse damage, was stimulus specific; these neurons were not protected against αSN-induced synapse damage. The protective effect of monoacylated PrP^C^ was long lasting and was related to the long half-life of monoacylated PrP^C^ in neurons. Although neurons decorated with monoacylated PrP^C^ bound similar amounts of Aβ as control neurons the Aβ did not cause synapse damage indicating that the presence of Aβ alone does not cause synapse damage and that synapse damage is mediated via specific mechanisms.

PrP^C^ has been associated with the activation of cPLA_2_, which occurs within rafts [11,38]. Some clues about how the binding of Aβ to PrP^C^ activates cPLA_2_ can be gathered from the prion literature, where aggregation of PrP^C^ caused synapse damage in neurons similar to that seen with aggregates of PrP^Sc^ [19].The observations that Aβ oligomers that can cross-link PrP^C^ are toxic, but Aβ monomers are not, indicate that the clustering of PrP^C^ is key to cell signaling and link prion and Alzheimer’s diseases to a common pathway leading to neurodegeneration. In this regard, it is of interest that in a transgenic mouse model of AD containing APPPS1^+^ Prnp°^/^° and crossed with mice producing anchorless PrP^C^ [39] the APPPS1-related suppression of LTP was inhibited; an effect that was independent of any effects upon the production of Aβ_42_ [40]. The oligomerization of GPI-anchored proteins stimulates raft formation [41] and the clustering of specific GPI anchors leads to activation of cPLA_2_ and synapse damage [19]. In this study, the presence of monoacylated PrP^C^ reduced Aβ-induced activation of cPLA_2_ in synapses as complexes formed by the aggregation of monoacylated PrP^C^ did not contain cPLA_2_.

## 5. Conclusions

We report that monoacylated PrP^C^ bound natural Aβ and that neurons decorated with monoacylated PrP^C^ were protected against Aβ-induced synapse damage. These studies support the hypothesis that the GPI anchor attached to PrP^C^ plays a role in mediating the effects of Aβ on neurons. The protective effect of monoacylated PrP^C^ was two fold: firstly, monoacylated PrP^C^ sequestered Aβ into cellular compartments not associated with cell signaling; and secondly, we demonstrate that monoacylated PrP^C^ reduced the Aβ-induced translocation and subsequent activation of cPLA_2_ that leads to synapse damage. Cell signaling by GPI-anchored proteins is a poorly understood process; proteins with modified GPI anchors may help explain this process.
